# Chronic Spontaneous Urticaria in the Era of Targeted Therapy: From Pathogenic Mechanisms to Precision Medicine

**DOI:** 10.3390/pharmaceutics18091169

**Published:** 2026-09-16

**Authors:** Klara Andrzejczak, Wiktor Witkowski, Joanna Górka-Dynysiewicz, Robert Pawłowicz, Agnieszka Kopeć

**Affiliations:** 1 Faculty of Medicine, Wroclaw Medical University, Wybrzeze L. Pasteura 1, 50-367 Wroclaw, Poland; klara.andrzejczak@student.umw.edu.pl (K.A.); wiktor.witkowski@student.umw.edu.pl (W.W.); 2 Department of Pharmaceutical Biochemistry, Faculty of Pharmacy, Wroclaw Medical University, 50-367 Wroclaw, Poland; 3 Clinical Department of Allergology and Internal Diseases, Institute of Internal Diseases, Wroclaw Medical University, 50-556 Wroclaw, Poland; robert.pawlowicz@umw.edu.pl (R.P.); agnieszka.kopec@umw.edu.pl (A.K.)

**Keywords:** chronic spontaneous urticaria (CSU), mast cells, immunopathogenesis, targeted therapy, biologic therapy, omalizumab, dupilumab, Bruton’s tyrosine kinase inhibitors, precision medicine

## Abstract

Chronic spontaneous urticaria (CSU) is a common and heterogeneous inflammatory skin disease characterized by recurrent wheals and/or angioedema for more than six weeks. Despite treatment with second-generation antihistamines, many patients remain symptomatic, highlighting the need for therapies targeting underlying pathogenic mechanisms. Aim: This review summarizes current knowledge on CSU immunopathogenesis and targeted therapies, with a focus on personalized medicine. Methods: A literature review was conducted, focusing on recent advances in CSU endotypes and clinical trial data on biologic, small-molecule, and other targeted therapies acting on key immunological pathways. Results: CSU is increasingly recognized as a heterogeneous disease with distinct immunological endotypes, including type I (autoallergic) and type IIb (autoimmune), which may influence treatment response. Omalizumab remains the mainstay of second-line therapy, although many patients do not achieve complete disease control. Emerging therapies, including anti-IL-4Rα monoclonal antibodies (dupilumab), Bruton’s tyrosine kinase inhibitors (e.g., remibrutinib), and anti-c-KIT monoclonal antibodies (e.g., barzolvolimab, briquilimab), have demonstrated promising efficacy. Some show a rapid onset of action, although preferential efficacy in specific CSU endotypes remains to be established. Several clinical and laboratory features, including total IgE, anti-TPO antibodies, ASST, basophil reactivity, basopenia, eosinopenia, and atopic comorbidities, have been investigated as potential markers of endotype and treatment response; however, their utility for treatment selection remains insufficiently validated. Conclusions: Advances in understanding CSU pathogenesis have enabled the development of targeted therapies that may improve disease control. Predictive biomarkers and endotypic characterization may support the future development of more personalized therapeutic strategies; however, clinically validated biomarkers for treatment selection remain limited, and prospective validation of biomarker-guided approaches is still needed.

## 1. Introduction

Chronic urticaria (CU) is one of the most common chronic skin diseases, affecting approximately 1% of the general population. It is broadly classified into two main forms: chronic spontaneous urticaria (CSU) and chronic inducible urticaria (CIndU). The inducible form, which accounts for approximately 20% of cases, is triggered by specific physical stimuli such as pressure, cold, heat, or vibration. In the remaining patients, symptoms occur without an identifiable trigger, thereby defining chronic spontaneous urticaria.

CSU is a complex disease driven by mast cell activation, characterized by the recurrent occurrence of wheals, angioedema, or both, persisting for more than six weeks [1,2,3,4]. The condition occurs more frequently in women, with the highest incidence observed in adulthood, particularly in the third to fifth decades of life. The increased incidence in this group may be related to the influence of sex hormones and autoimmune mechanisms [5,6].

In most patients, symptoms persist for more than a year, often exhibiting a recurrent course [4]. The course of CSU is variable and difficult to predict, and spontaneous remission rates vary considerably across studies. These differences likely arise from heterogeneous definitions of remission, variations in population characteristics, and differences in assessment time points [7,8]. CSU symptoms result from mast cell degranulation and the release of inflammatory mediators, which drive the development of skin lesions [9].

Patients with CSU have been shown to exhibit an increased prevalence of atopic diseases, including atopic dermatitis, allergic rhinitis, asthma, and food allergies [10,11,12,13]. Furthermore, CSU is frequently associated with autoimmune diseases, particularly autoimmune thyroid diseases, which are among the most common comorbidities in this population. The coexistence of vitiligo, rheumatoid arthritis, Sjögren’s syndrome, type 1 diabetes, and celiac disease has also been reported [13,14,15,16,17,18]. Patients also exhibit an increased prevalence of metabolic syndrome, sleep disturbances, and psychiatric disorders, including anxiety and depression, highlighting the complex and multidimensional nature of the disease. The recurrent and unpredictable course of the disease, substantial symptom burden, and limited effectiveness of standard treatment represent a major clinical challenge and highlight the need for new targeted therapies [1,4,19].

This review summarizes current and emerging treatment strategies for chronic spontaneous urticaria, with particular emphasis on targeted therapies directed at specific pathogenic mechanisms and their potential role in enabling more individualized treatment approaches. Special attention is given to recently approved and investigational agents, including biologic therapies, small-molecule inhibitors, and novel mast cell-directed strategies, and to linking their mechanisms of action with the diverse immunological pathways underlying CSU.

Despite the availability of standard treatments, a substantial proportion of patients remain symptomatic, and the heterogeneity of CSU endotypes is associated with variable therapeutic responses. These challenges highlight the clinical need for mechanism-based therapeutic strategies and support the ongoing transition of CSU management toward more precise, personalized treatment.

## 2. Materials and Methods

This review was designed as a narrative literature review based on a structured analysis of current evidence concerning chronic spontaneous urticaria (CSU) endotypes, immunopathogenesis, and targeted therapies, with particular emphasis on their potential role in mechanism-based precision medicine. The main objective of the literature search was to identify relevant studies describing mast cell activation pathways, biomarkers of treatment response, and biologic, small-molecule, and other emerging therapies targeting key immunological mechanisms in CSU.

A literature search was conducted using major biomedical and multidisciplinary databases, including PubMed, Scopus, Web of Science, Embase, and Google Scholar. The search focused primarily on publications from the last decade in order to reflect recent advances in CSU pathogenesis, endotyping, biomarker research, and targeted therapy. However, earlier landmark studies, key guideline documents, and pivotal clinical trials were also included when they provided essential background information, historical context, clinically relevant definitions, or data on established therapeutic strategies. The final literature search was conducted in August 2026 to update the evidence base before manuscript finalization.

The search strategy included combinations of the following keywords and Medical Subject Headings (MeSH), where applicable: “chronic spontaneous urticaria”, “CSU”, “chronic urticaria”, “mast cells”, “basophils”, “autoallergy”, “autoimmune urticaria”, “type I autoallergic CSU”, “type IIb autoimmune CSU”, “IgE”, “FcεRI”, “omalizumab”, “dupilumab”, “IL-4”, “IL-13”, “Bruton’s tyrosine kinase”, “BTK inhibitors”, “remibrutinib”, “rilzabrutinib”, “fenebrutinib”, “c-KIT”, “barzolvolimab”, “briquilimab”, “MRGPRX2”, “tezepelumab”, “IL-17”, “biomarkers”, “endotypes”, “precision medicine”, and “targeted therapy”.

The review prioritized original clinical trials, extension studies, real-world evidence, translational studies, mechanistic investigations, international guidelines, and regulatory documents relevant to targeted therapies in CSU. Review articles were used mainly to identify additional primary sources and to provide background context. Studies were considered particularly relevant if they addressed CSU immunopathogenesis, therapeutic mechanisms of action, clinical efficacy, safety, predictors of treatment response, or potential biomarkers useful for patient stratification. Potentially relevant records were assessed based on their titles and abstracts, followed by full-text evaluation when appropriate. Non-English publications, conference abstracts without sufficient clinical or methodological detail, duplicate reports, and studies not directly related to CSU or targeted treatment mechanisms were not included as core evidence.

The strength and quality of evidence were considered qualitatively according to study design and methodological characteristics. Randomized controlled trials and prospective clinical studies were given greater weight when assessing therapeutic efficacy, whereas observational and real-world studies were primarily considered for treatment-response patterns and clinical applicability. Mechanistic and translational studies were used mainly to support biological rationale and endotype interpretation.

Given the broad scope of the topic and the substantial heterogeneity of the available evidence, including differences in study design, patient populations, therapeutic interventions, biomarkers, and reported outcomes, a quantitative meta-analysis was not performed. Findings were instead synthesized narratively and organized thematically according to major pathogenic and therapeutic domains: CSU endotypes and mast cell activation pathways, standard management with H1-antihistamines, anti-IgE therapy, type 2 inflammation blockade, BTK inhibition, c-KIT-targeted therapy, MRGPRX2 pathway modulation, and other cytokine-targeted approaches. Particular attention was given to the relationship between pathogenic mechanisms and therapeutic targets, with the aim of highlighting the potential role of endotype-driven treatment selection in CSU.

## 3. Pathogenesis of Chronic Spontaneous Urticaria and Therapeutic Targets

Chronic spontaneous urticaria is a complex and heterogeneous disease with an incompletely understood pathogenesis [20]. Mast cells play a central role in its development, and their activation leads to the release of a range of proinflammatory mediators, including histamine, which is responsible for pruritus and wheal formation, as well as platelet-activating factor (PAF) and cytokines. These mediators stimulate sensory nerves, induce vasodilation, and promote the recruitment of inflammatory cells [1,21].

CSU exhibits features of a type 2-driven immune response; however, its pathogenesis remains complex and involves both autoallergic (IgE-dependent) and autoimmune (IgG-dependent) mechanisms. Activation of epithelial cells can lead to the release of alarmins (TSLP, IL-25, IL-33), which stimulate type 2 innate lymphoid cells (ILC2s) and promote Th2 polarization of T cells. Consequently, cytokines such as IL-4, IL-5, and IL-13 are produced, inducing class switch recombination in B cells toward IgE production. In parallel, IgG autoantibodies directed against FcεRI or IgE may also contribute to mast cell activation. Together, these mechanisms can trigger mast cell degranulation [22,23,24,25].

Mast cell activation in CSU can occur through a variety of pathways, including both immunologic (IgE-dependent and IgE-independent) and non-immunologic mechanisms, such as direct degranulation triggered by chemical or physical stimuli. Current evidence suggests that autoimmune processes play a key role in a substantial proportion of patients [26,27,28].

This mechanistic diversity is reflected in the heterogeneity of CSU and has led to the recognition of two main immunological endotypes. These endotypes may overlap, with features of both type I autoallergic and type IIb autoimmune CSU reported in approximately 7% of patients, while others may not be readily classified within either endotypic pattern [28,29].

Type I (autoallergic) CSU is characterized by IgE autoantibodies directed against self-antigens, which can activate mast cells through the high-affinity IgE receptor (FcεRI). Several autoantigens have been identified, including thyroid peroxidase (TPO), double-stranded DNA (dsDNA), and IL-24. Type I autoallergic mechanisms appear to occur in a substantial proportion of patients with CSU, although reported prevalence varies depending on the autoantigen investigated and the methods used to detect autoreactive IgE. Patients with this endotype more frequently exhibit atopic features and generally have normal or elevated total IgE levels [28,30].

Type IIb autoimmune CSU is driven by functional IgG autoantibodies directed against IgE and/or its high-affinity receptor (FcεRI), which promote mast cell and basophil activation, leading to degranulation and subsequent urticaria symptoms. However, the identification and reported prevalence of this endotype depend strongly on the diagnostic criteria applied. Strict type IIb aiCSU is defined by the concurrent presence of IgG autoantibodies against IgE and/or FcεRI, positive basophil reactivity assessed using the basophil activation test (BAT) and/or basophil histamine release assay (BHRA), and a positive autologous serum skin test (ASST). In the PURIST study, approximately 8% of patients fulfilled all three criteria for strict type IIb aiCSU [31].

Several clinical and laboratory features, including low total IgE levels, elevated anti-TPO IgG, basopenia, eosinopenia, and isolated ASST positivity, are associated with this endotype. However, when present in isolation, these findings should be regarded as supportive or surrogate markers rather than definitive diagnostic criteria for strict type IIb aiCSU. This endotypic profile has also been associated with higher disease activity, a greater burden of autoimmune comorbidities, and a poorer or delayed response to omalizumab [32,33,34,35].

Although several clinical and laboratory markers have been associated with specific CSU endotypes and treatment responses, their current role remains primarily supportive and predictive rather than prescriptive. At present, no biomarker-guided therapeutic algorithm has been sufficiently validated in prospective studies to enable reliable endotype-driven treatment selection in routine clinical practice. Therefore, these markers should be interpreted in conjunction with the overall clinical phenotype, disease characteristics, and treatment history rather than used as stand-alone determinants of therapeutic choice [1,28,36].

Mas-related G protein-coupled receptor X2 (MRGPRX2)-mediated mast cell activation may also play an important role in the pathogenesis of CSU. The expression of this receptor is upregulated in patients with CSU compared with healthy individuals [37,38].

Stem cell factor (SCF) is a key regulator of mast cell differentiation, survival, and activation, signaling through the c-KIT receptor (CD117) [39,40]. Mast cell activation is also regulated by intracellular signaling pathways in which Bruton’s tyrosine kinase (BTK) plays a central role. BTK is involved in FcεRI- and B cell receptor (BCR)-mediated signaling, thereby regulating mast cell degranulation and autoantibody production [41].

Additional immune cells and mediators, including eosinophils and cytokines such as IL-17 and IL-23, may also contribute to CSU pathogenesis.

A deeper understanding of these mechanisms has enabled the development of novel targeted therapies aimed at specific pathogenic pathways. However, the heterogeneity of these mechanisms is reflected in variable treatment responses, underscoring the need for a more personalized, mechanism-based approach to therapy selection in CSU [1,42,43].

## 4. Therapeutic Management of Chronic Spontaneous Urticaria

CSU management aims not only to alleviate distressing symptoms but also to achieve complete disease control, long-term remission, and, where possible, sustained disease control after treatment discontinuation.

In patients with CSU, disease activity and level of control should be regularly evaluated using validated tools such as the Urticaria Activity Score (UAS), including its weekly version (UAS7), and the Urticaria Control Test (UCT). Complete disease control is defined as UAS7 = 0 and UCT = 16, corresponding to the complete resolution of wheals and pruritus [1,44].

Current international recommendations for CSU management include second-generation H1-antihistamines as first-line therapy at standard doses, with updosing up to fourfold in patients with insufficient disease control. In patients who remain symptomatic despite high-dose second-generation H1-antihistamines, add-on therapy targeting key pathogenic pathways should be considered. Omalizumab remains the most established option and is strongly recommended as the next step in the treatment algorithm, based on extensive evidence supporting its efficacy and favorable safety profile. The most recent international guideline has also incorporated dupilumab and remibrutinib as add-on treatment options for patients with CSU unresponsive to high-dose second-generation H1-antihistamines, although these recommendations are supported by a lower strength of consensus than that for omalizumab. Dupilumab provides an additional biologic strategy through IL-4/IL-13 pathway inhibition, whereas remibrutinib represents the first oral BTK inhibitor incorporated into the therapeutic algorithm. Beyond these recently incorporated options, several additional targeted therapies are currently under clinical investigation, further expanding the therapeutic landscape of CSU [1].

This growing therapeutic diversity may facilitate treatment individualization based on clinical characteristics, previous treatment response, comorbidities, route-of-administration preferences, and safety considerations. However, current guidelines do not establish a validated biomarker- or endotype-based sequence for selecting among these therapies [1].

In patients with severe CSU refractory to licensed treatments, or when these treatments are unavailable, ciclosporin may be considered, although its use in urticaria remains off-label and is limited by a less favorable long-term safety profile. As an immunomodulatory calcineurin inhibitor, ciclosporin suppresses T-cell activation by reducing the production of cytokines such as IL-2, IL-3, IL-4, and TNF-α, thereby attenuating the immune response, while also directly reducing mast cell mediator release. Current guidelines further indicate that its efficacy may be particularly relevant in selected patients with type IIb autoimmune CSU, especially those with low total IgE levels and other features suggestive of autoimmunity [1,45,46].

### H1-Antihistamines as First-Line Therapy

H1-antihistamines are the mainstay of treatment for chronic spontaneous urticaria. Acting as inverse agonists at the H1 receptor, they inhibit the effects of histamine released from activated mast cells and modulate the associated inflammatory response.

First-generation antihistamines are not recommended in either adults or children due to their unfavorable safety profile, including sedative and anticholinergic effects, disruption of REM sleep, and a high potential for drug interactions.

Second-generation H1-antihistamines are preferred in clinical practice due to their minimal sedative effects, lack of significant anticholinergic activity, and reduced risk of drug interactions. They also have a longer half-life. Despite their more favorable safety profile, some second-generation agents, such as astemizole and terfenadine, have been withdrawn due to their potential cardiotoxicity.

However, the effectiveness of antihistamine treatment remains limited [1,47,48,49,50,51,52]. A substantial proportion of patients remain symptomatic despite treatment with second-generation antihistamines, even at increased doses [53].

Real-world data further highlight the limited effectiveness of antihistamine therapy. In a study by Bernstein et al., disease control remained inadequate in 84% of patients despite switching between H1-antihistamines and dose escalation, and approximately one-quarter required subsequent lines of therapy.

These findings underscore the need for more effective, mechanism-based treatment strategies, as antihistamines primarily provide symptomatic relief without directly targeting the underlying pathogenic pathways [54].

## 5. Targeted and Advanced Therapies in Chronic Spontaneous Urticaria

The growing understanding of the underlying pathogenic mechanisms of CSU has enabled the development of targeted therapies directed at key immunological pathways. In clinical practice, treatment strategies primarily focus on inhibiting the effects of mast cell mediators, limiting their release, and blocking mast cell activation.

Emerging therapies are increasingly focused on more selective modulation of the immune response, including modulation of type 2 inflammation, enhancement of inhibitory signaling in mast cells, and reduction in mast cell numbers, potentially leading to more durable disease control. Numerous agents targeting different elements of CSU pathogenesis are currently at various stages of development and represent a promising expansion of available therapeutic options, particularly for patients who do not respond to standard treatment. These advances further support a more individualized, mechanism-based approach to therapy selection based on the dominant disease pathways [1].

**Figure 1:**
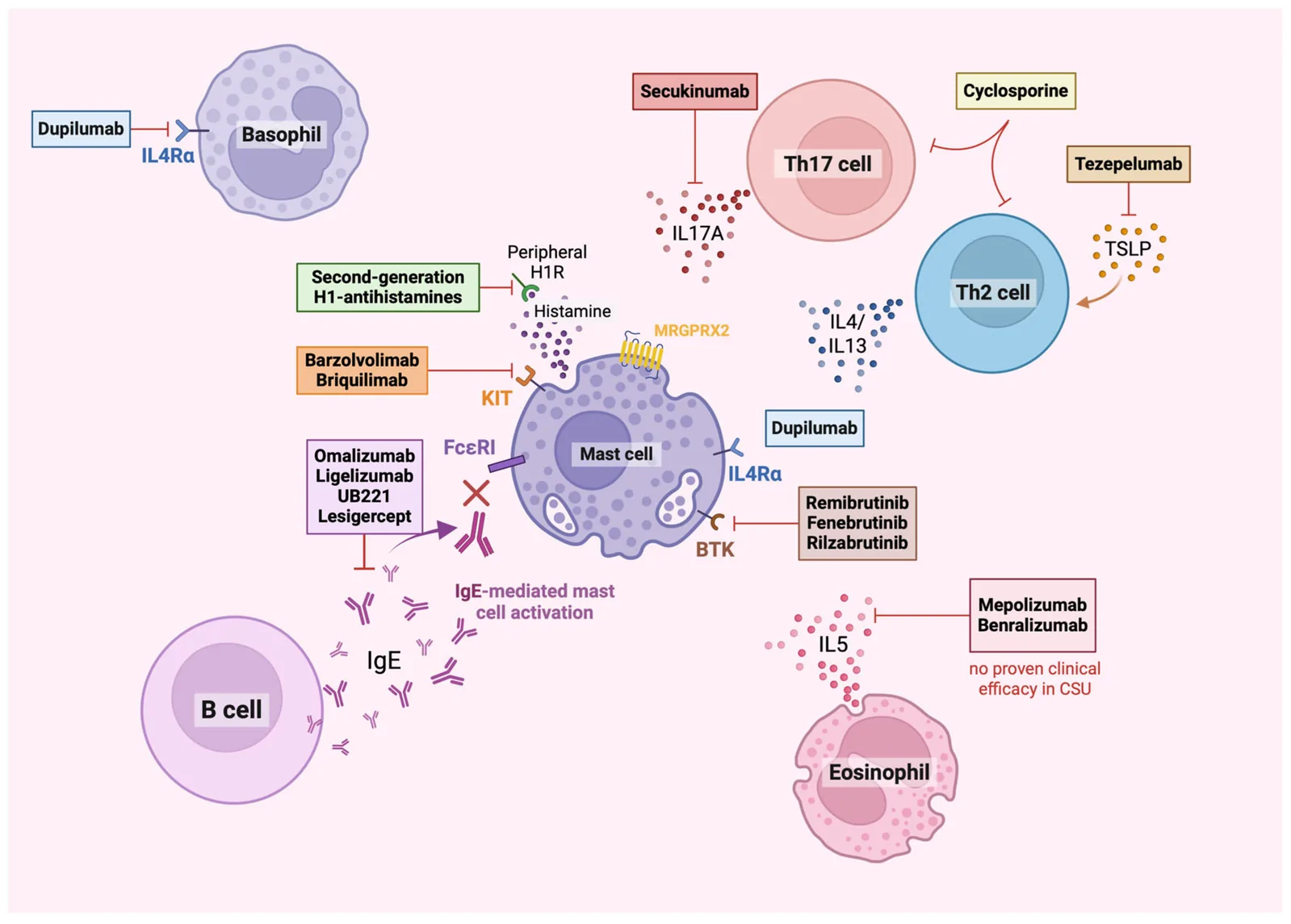
Mechanism-Based Targeting in Chronic Spontaneous Urticaria: Pathways and Therapies. Created in BioRender. Andrzejczak, K. (2026) https://BioRender.com/qtihdze.

An overview of the key pathogenic pathways and their corresponding therapeutic targets in CSU is provided in Figure 1.

### 5.1. Biologic Therapies Targeting Key Immunologic Pathways in CSU

#### 5.1.1. Omalizumab: Targeting the IgE Pathway

Omalizumab was approved in 2014 by both the U.S. Food and Drug Administration (FDA) and the European Medicines Agency (EMA) for the treatment of chronic spontaneous urticaria in patients aged ≥12 years. It was the first biologic approved for this indication.

It is a recombinant, humanized monoclonal IgG antibody with high affinity for free IgE. By binding free IgE, it reduces serum IgE levels, resulting in downregulation of FcεRI expression and decreased IgE-binding capacity on the surface of mast cells and basophils [55,56,57]. Additionally, reduced FcεRI expression on mast cells, epidermal dendritic cells, and Langerhans cells results from the degradation of unbound FcεRI, which is not stabilized by IgE binding [58,59]. Omalizumab has also been shown to normalize gene expression within CSU skin lesions [60].

The standard dose of omalizumab is 300 mg every 4 weeks, regardless of baseline IgE levels. In patients with an insufficient response, off-label updosing may be considered by increasing the dose and/or shortening the dosing interval, up to a maximum of 600 mg every 2 weeks, as supported by current international recommendations and real-world evidence [1,61].

The efficacy and safety of omalizumab have been demonstrated in numerous randomized clinical trials and supported by real-world evidence (RWE). A clinical response is observed in approximately 45% of patients with CSU refractory to antihistamine therapy, with complete disease control achieved in around 30% of patients [57,61,62,63,64,65,66,67].

Nevertheless, the response to therapy is variable and depends on multiple clinical and immunological factors. Predictors of a poorer or delayed response to treatment include low baseline total IgE levels, a positive result of the ASST, antinuclear antibody (ANA) positivity, eosinopenia, basopenia, elevated C-reactive protein (CRP) levels, and the presence of concomitant autoimmune diseases [34,68,69,70,71].

Furthermore, UAS7 and the Dermatology Life Quality Index (DLQI) are well-established, validated patient-reported outcome measures used to assess disease activity and health-related quality of life, respectively. In contrast, immunological parameters such as IL-17 levels have been investigated as potential biomarkers of treatment response [66].

Among the available laboratory parameters, baseline total IgE has received particular attention and may have clinically relevant prognostic value for omalizumab response. In a study by Ertas et al., low baseline total IgE levels (<43 IU/mL) were associated with a 33% risk of lack of response within the first 12 weeks, compared with 5% in patients with IgE levels ≥43 IU/mL [68]. In contrast, levels >100 IU/mL were more frequently observed in early responders, suggesting that higher pretreatment IgE concentrations may be associated with a more favorable therapeutic response [72,73,74].

Notably, the therapeutic effect may be delayed. A post hoc analysis of the phase IV (XTEND-CIU) study demonstrated that so-called late responders, defined as patients achieving UAS7 ≤ 6 not until week 24, accounted for approximately 16% of the population. These patients were more likely to have low baseline IgE levels, higher body mass index, poorer disease control, and a younger age at treatment initiation [75].

The disease endotype also plays an important role. Patients with the autoallergic endotype, often associated with concomitant atopic diseases, appear to respond particularly well to omalizumab, whereas those with the autoimmune endotype may exhibit a weaker response [34,72,73,76].

Omalizumab has a favorable safety profile. Serious adverse events, including anaphylaxis, are rare [77,78], and transient hair loss, including alopecia areata, has been occasionally reported [79,80].

Despite the high efficacy of omalizumab, a significant proportion of patients do not achieve complete disease control, underscoring the need for new therapeutic options. A network meta-analysis of randomized controlled trials demonstrated that omalizumab at a dose of 300 mg exhibited the highest effect sizes across major clinical outcomes in CSU, including reductions in disease activity, itch severity, and achievement of symptom control. However, newer agents such as remibrutinib and dupilumab have also shown substantial efficacy.

These findings support the potential for a more individualized approach to CSU management based on underlying pathogenic mechanisms and patient characteristics, although validated biomarker-guided treatment selection remains unavailable. Further research should focus on defining the optimal place of these emerging therapies within the therapeutic algorithm and identifying patient subgroups most likely to benefit from them [81].

#### 5.1.2. Emerging Anti-IgE Therapies and Biosimilars

Other therapies targeting the IgE pathway include monoclonal antibodies with modified properties as well as omalizumab biosimilars currently under development for the treatment of CSU.

Ligelizumab is a humanized IgG1 monoclonal antibody with high affinity for IgE, nearly 50-fold higher than that of omalizumab. Despite promising results from phase II studies and demonstrated efficacy versus placebo, phase III PEARL 1 and 2 trials failed to confirm its superiority over omalizumab, which limited its further development in the treatment of CSU [82,83,84,85].

UB-221 is a humanized monoclonal antibody with greater IgE-neutralizing activity than omalizumab. It binds IgE, forming oligomeric complexes that interact with the low-affinity IgE receptor CD23 (FcεRII), which is involved in the regulation of mast cell activity. UB-221 is currently in phase II clinical trials [86].

Lesigercept (YH35324) is a hybrid protein containing the extracellular domain of FcεRIα, designed to capture free IgE and antibodies targeting FcεRIα. A phase I study has been completed, and enrollment in a phase II trial is currently planned in patients with CSU refractory to antihistamines [87,88].

Omalizumab biosimilars represent an important extension of these strategies. The European Medicines Agency has approved CT-P39 (Omlyclo, Celltrion) in the European Union, demonstrating comparable structural, biological, and clinical properties to omalizumab [89]. In China, SYN008 has been approved, while additional biosimilars (GBR-310 and BP-11) are currently under development [90,91].

#### 5.1.3. Dupilumab: Modulation of IL-4/IL-13 Signaling

Key components of CSU pathophysiology include mast cells, eosinophils, and basophils, whose activity can be modulated by the type 2 immune response. The cytokines IL-4 and IL-13 play a key role in this response, contributing to the exacerbation of disease symptoms through direct and indirect activation of effector cells, enhanced migration of inflammatory cells to the skin, regulation of IgE production, and sensitization of nerve fibers, thereby providing a rationale for therapies targeting this pathway [21,51,92,93].

Dupilumab is a fully human monoclonal antibody directed against the IL-4 receptor alpha subunit (IL-4Rα), a shared receptor component for IL-4 and IL-13. By blocking this subunit, it inhibits signaling of both cytokines. Dupilumab has demonstrated efficacy in multiple diseases associated with type 2 inflammation [92,94,95].

Dupilumab was first approved in 2017 for the treatment of atopic dermatitis and subsequently received indications for asthma, chronic rhinosinusitis with nasal polyps, eosinophilic esophagitis, prurigo nodularis, and chronic obstructive pulmonary disease (COPD). In April 2025, the FDA approved dupilumab for the treatment of CSU in patients aged ≥12 years, while in February 2026, the EMA recommended an extension of the indication to younger patients aged 2-12 years, which would make dupilumab the first biologic therapy in this population [96].

In CSU, dupilumab exerts its therapeutic effects by decreasing IgE production, inhibiting eosinophil infiltration, and reducing mast cell activation.

The efficacy of dupilumab in CSU has been evaluated in the phase III LIBERTY-CSU CUPID program. The pivotal CUPID-A and CUPID-C trials included a total of 289 antihistamine-refractory, omalizumab-naive patients. Dupilumab significantly reduced disease activity, resulting in reductions in pruritus severity and urticaria activity compared with placebo, as confirmed in a pooled analysis of both trials. Dupilumab reduced UAS7 independently from total IgE. These results indicate clinically significant efficacy of dupilumab and its favorable safety profile [92,95].

In contrast, CUPID-B, which included patients with omalizumab intolerance or an inadequate response to anti-IgE therapy, did not meet its primary endpoint, with limited efficacy observed in this population [95].

Dupilumab has a favorable safety profile, comparable to that of placebo, with the most common adverse events including injection site reactions and upper respiratory tract infections, including nasopharyngitis [97,98].

The recommended dosing regimen of dupilumab is based on body weight. Patients weighing ≥60 kg receive a loading dose of 600 mg followed by 300 mg every 2 weeks, whereas those weighing ≥30 kg receive a loading dose of 400 mg followed by 200 mg every 2 weeks. In patients weighing ≥15 kg, a loading dose of 400 mg is administered, followed by 200 mg every 4 weeks [1].

The limited efficacy of dupilumab in patients with an inadequate response to omalizumab suggests that factors beyond type 2 inflammation may contribute to treatment resistance in this subgroup, although the underlying mechanisms remain to be established. Accordingly, dupilumab may represent a therapeutic alternative in selected patients, particularly in cases of omalizumab intolerance or unavailability, and may be particularly relevant in patients with concomitant type 2 inflammatory diseases. The variable efficacy of dupilumab depending on phenotype and prior treatment response indicates that its optimal use may require a more personalized therapeutic approach, taking into account the dominant pathogenetic mechanisms in individual patients [1,95].

### 5.2. Small Molecule Therapies Targeting Intracellular Signaling Pathways in CSU: Bruton’s Tyrosine Kinase Inhibitors

Both autoallergic and autoimmune mechanisms lead to the activation of mast cells and basophils through cross-linking of FcεRI and activation of intracellular signaling pathways. Bruton’s tyrosine kinase (BTK) plays a key role in these processes as a central component of the FcεRI-dependent signaling cascade and a regulator of signaling pathways in effector cells and B lymphocytes. Activation of this pathway represents one of the main mechanisms initiating mast cell degranulation and the development of the inflammatory response.

BTK inhibition allows for simultaneous inhibition of mast cell and basophil degranulation, reduction in de novo cytokine synthesis in mast cells, and suppression of activation and differentiation of autoreactive B lymphocytes, leading to decreased autoantibody production. Consequently, BTK inhibitors (BTKi) demonstrate a dual mechanism of action, combining inhibition of effector cell activation with modulation of the B-cell-mediated autoimmune response. The involvement of BTK in both FcεRI-dependent signaling and B-cell-mediated pathways provides a strong mechanistic rationale for BTK inhibition in CSU. However, this mechanistic rationale does not by itself establish preferential clinical efficacy in specific CSU endotypes or in patients with an inadequate response to IgE-targeted therapies [41,51,99,100,101].

New, more selective BTK inhibitors with improved safety profiles have been developed compared with earlier generations, whose use was limited. Their efficacy and safety are currently being evaluated across different phases of clinical development in patients with CSU [100,102].

Among these, remibrutinib, an oral, highly selective BTK inhibitor, is of particular interest [99,100]. In September 2025, the FDA approved it as the first oral targeted therapy for CSU in adult patients who remain symptomatic despite H1-antihistamine treatment [103,104]. In April 2026, remibrutinib (Rhapsido) received marketing authorization in the European Union for the treatment of CSU in adult patients with inadequate disease control despite antihistamine treatment [105]. This approval represents a significant therapeutic advance, offering a convenient oral alternative to previously used parenterally administered biologic therapies [104].

This decision was based on the results of two phase III clinical trials REMIX 1 and REMIX 2, enrolling over 900 patients with CSU uncontrolled despite antihistamine therapy. Remibrutinib demonstrated a significant improvement in symptom control (reduction in UAS7 score) during the 12-week primary analysis period compared with placebo, and this effect was maintained through week 24 [106].

Data from extended follow-up of up to 52 weeks further indicate sustained efficacy and a consistent, favorable safety profile for remibrutinib [107]. The incidence of adverse events was comparable to that of placebo, although nasopharyngitis, upper respiratory tract infections, and petechiae were reported slightly more frequently.

Remibrutinib is characterized by a rapid onset of action, with improvements in UAS7 observed as early as week 1 [108,109,110]. Beyond these clinical observations, experimental studies have shown that remibrutinib inhibits mast cell and basophil activation induced by IgE cross-linking as well as activation triggered by sera from patients with chronic urticaria. Importantly, these inhibitory effects were observed independently of baseline FcεRI expression and clinical responsiveness to omalizumab. However, these mechanistic findings do not establish preferential clinical efficacy in omalizumab nonresponders or in specific CSU endotypes [111].

In addition to remibrutinib, other BTK inhibitors have also been investigated. Fenebrutinib was evaluated in a small cohort of patients with chronic spontaneous urticaria, in whom rapid improvement in disease activity was observed, although the study was discontinued due to adverse events, including elevated liver enzyme levels. These findings provide preliminary evidence of clinical activity but are insufficient to establish its efficacy or safety relative to more extensively studied BTK inhibitors [112].

Rilzabrutinib, a highly selective, reversible BTK inhibitor with a prolonged duration of action, has been approved by the FDA for the treatment of chronic immune thrombocytopenia and is currently being investigated in patients with CSU. In the 52-week phase II RILECSU randomized clinical trial in patients with CSU inadequately controlled by antihistamines, rilzabrutinib 1200 mg/day was associated with rapid improvement in disease activity and itch, with effects observed as early as week 1, and significantly improved symptom control and itch severity compared with placebo. Furthermore, treatment led to a reduction in IgG autoantibody levels, including anti-TPO and anti-FcεRI. Rilzabrutinib was well tolerated, with a slightly higher incidence of adverse events such as headache, vomiting, and diarrhea [113,114].

TAS5315, another BTK inhibitor in clinical development for the treatment of autoimmune and allergic diseases, including chronic spontaneous urticaria, is also under investigation [115].

Overall, the clinical evidence for BTK inhibitors in CSU remains heterogeneous, with the most mature evidence currently available for remibrutinib, supported by phase III randomized trials and extended follow-up. In contrast, rilzabrutinib is supported by phase II randomized data, while evidence for fenebrutinib and other investigational BTK inhibitors remains more limited. Although the class has a strong mechanistic rationale and several agents demonstrate rapid clinical activity, current evidence does not allow reliable comparison of efficacy across individual BTK inhibitors or confirm preferential benefit in specific patient populations or CSU endotypes. Further comparative and longer-term studies are needed to clarify the relative efficacy, safety, durability, and optimal positioning of individual BTK inhibitors within the CSU treatment algorithm [106,107,108,109,110,111,112,113,114,115].

### 5.3. Novel Targets Beyond Established Pathways in CSU

#### 5.3.1. c-KIT Inhibition: Targeting Mast Cell Survival in CSU

Stem cell factor (SCF) plays a crucial role in mast cell differentiation and survival. In this context, c-KIT receptor blockade represents a promising therapeutic strategy in CSU. Targeting mast cell survival represents a distinct approach compared with current strategies, which primarily focus on inhibiting mast cell activation [39,40].

Barzolvolimab is a highly specific humanized monoclonal antibody that binds the extracellular domain of c-KIT receptor on mast cells. By blocking c-KIT, it inhibits SCF-dependent signaling, resulting in mast cell apoptosis and a reduction in their number and activity in tissues.

Phase 1b and 2 clinical trials demonstrated that barzolvolimab was associated with a rapid and significant reduction in disease activity, with high rates of clinical response and remission. Clinical effects were observed as early as the first week of treatment and were sustained during treatment with comparable responses in both patients previously treated with omalizumab and omalizumab-naive patients. Up to 74% of patients achieved well-controlled disease (UAS7≤6) with barzolvolimab at week 52. Available data indicate that barzolvolimab was generally well tolerated, with the most commonly reported adverse events being neutrophil count decrease and hair color changes [116,117].

Phase III studies EMBARQ-CSU 1 and EMBARQ-CSU 2 are currently underway to assess the efficacy and safety of barzolvolimab in adult patients with CSU inadequately controlled with antihistamine therapy [118,119].

Another monoclonal antibody with a similar mechanism of action is briquilimab, also targeting the c-KIT receptor. In the phase 1b/2a BEACON study, its efficacy, safety, and tolerability are being evaluated in patients with CSU, with further studies planned to assess its long-term efficacy and safety [120].

Taken together, the available evidence for c-KIT-targeted therapies in CSU remains at an earlier stage of development than that for established treatments. Barzolvolimab has demonstrated promising clinical activity in phase 1b/2 studies, including responses in both omalizumab-experienced and omalizumab-naive patients, but its efficacy and safety require confirmation in ongoing phase III trials. Evidence for briquilimab is currently limited to an early-phase study. Although c-KIT inhibition represents a mechanistically distinct approach targeting mast cell survival, current evidence is insufficient to establish its comparative efficacy, long-term safety, or preferential benefit in specific CSU endotypes. Further phase III and long-term comparative studies are needed to determine the optimal positioning of c-KIT-targeted therapies in CSU [116,117,118,119,120].

#### 5.3.2. MRGPRX2 Pathway Inhibition

Studies on EP262, an MRGPRX2 receptor inhibitor, have been terminated due to safety concerns [121]. Another compound, EVO756, a selective oral MRGPRX2 inhibitor, is currently in phase II clinical trials. However, data are currently insufficient to evaluate the efficacy of this therapeutic approach [122]. Thus, the MRGPRX2 pathway remains an investigational therapeutic target in CSU, with its clinical efficacy, safety, and optimal therapeutic positioning yet to be determined.

#### 5.3.3. Cytokine-Targeted Therapies in CSU

Tezepelumab is a monoclonal antibody directed against thymic stromal lymphopoietin (TSLP), an epithelial alarmin. By blocking TSLP binding to its receptor, it inhibits activation of type 2 inflammatory pathways, including Th2 cytokine production. Tezepelumab is approved for the treatment of severe asthma and chronic rhinosinusitis with nasal polyps [123,124,125].

The phase IIb INCEPTION study, which included patients with CSU uncontrolled despite H1-antihistamine treatment, did not meet its primary endpoint of a significant reduction in disease activity as measured by UAS7 at week 16. Tezepelumab did not demonstrate superiority over placebo.

Subgroup analyses suggested potential benefit in patients previously untreated with anti-IgE therapy, particularly those with lower baseline IgE levels and longer disease duration. In this population, delayed but sustained improvement in disease activity was observed up to week 32, even after treatment discontinuation.

Despite not achieving the primary endpoint, the study findings suggest a gradual and sustained reduction in CSU activity, potentially reflecting a delayed effect of TSLP blockade. The safety profile of the drug was comparable to that of placebo [123].

Secukinumab is a monoclonal antibody directed against IL-17A, which plays a significant role in the pathogenesis of many autoimmune diseases and may also be involved in the development of CSU. In a small clinical study, its efficacy was evaluated in eight patients with severe, refractory disease, unresponsive to H1-antihistamines and omalizumab. All patients demonstrated significant improvement in disease activity, although the therapeutic effect developed gradually [42]. Further large-scale randomized controlled trials are warranted to confirm the efficacy of secukinumab in patients with CSU.

Although CSU is primarily a mast cell-driven disease, eosinophils may be present as a secondary component of the inflammatory response. However, the available evidence for therapies targeting the IL-5 pathway in CSU remains limited and differs between agents. In the randomized, double-blind, placebo-controlled phase IIb ARROYO trial, benralizumab did not demonstrate significant clinical benefit compared with placebo. In contrast, mepolizumab was evaluated in an open-label, single-arm exploratory study in patients with antihistamine-refractory CSU, in which treatment was not associated with clinical responses, as assessed by UAS7 and Urticaria Control Test (UCT) scores, or with laboratory responses related to eosinophilia [43,126,127].

Current evidence for cytokine-targeted therapies in CSU is heterogeneous and generally limited, reflecting differences in study design, therapeutic targets, and patient populations. While some subgroup analyses suggest potential benefit in selected patients, these findings require prospective confirmation. At present, the available evidence is insufficient to establish a clear role for cytokine-targeted therapies in routine CSU management or to define specific patient populations most likely to benefit.

The mechanism-based therapies discussed in this review are summarized in Table 1, with emphasis on their molecular targets, clinical status, key evidence, and current limitations.

### 5.4. Toward Precision Medicine in CSU

#### 5.4.1. Comparative Evidence and Therapeutic Positioning of Targeted Therapies

The individual targeted therapies discussed above differ in their mechanisms of action, level of clinical evidence, efficacy, safety, and stage of development. This section provides a comparative synthesis of the available evidence, with a particular focus on therapies supported by the most substantial clinical evidence, including comparative efficacy, speed of response, and factors relevant to their potential therapeutic positioning.

Comparative evidence from recent network meta-analyses and indirect treatment comparisons suggests clinically meaningful efficacy across several targeted therapies in antihistamine-refractory CSU. Omalizumab 300 mg and oral remibrutinib at doses of 35 mg, 25 mg, or 10 mg have demonstrated efficacy in patients with antihistamine-refractory CSU, with remibrutinib showing a favorable balance between efficacy and safety and providing rapid disease control. Its oral route of administration may also represent a convenient treatment option for patients with moderate-to-severe CSU who prefer oral therapy [128].

In another network meta-analysis of randomized controlled trials comparing the efficacy of omalizumab, dupilumab, and remibrutinib in CSU, omalizumab showed the strongest efficacy across major clinical outcomes, followed by remibrutinib. Both remibrutinib and dupilumab appeared generally effective, highlighting their potential as treatment options for patients who do not achieve adequate disease control with standard therapies. However, dupilumab was associated with sustained improvement in itch, although its clinical efficacy appeared to develop more gradually [81].

A matching-adjusted indirect comparison also reported greater improvements in disease activity and itch severity with omalizumab compared with dupilumab [129]. A network meta-analysis evaluating time to meaningful clinical response also found statistically significant reductions compared with placebo for omalizumab, dupilumab, and ligelizumab [130].

These findings should therefore be interpreted cautiously because of differences in study populations, dosing regimens, endpoints, and treatment duration. Direct head-to-head randomized trials are needed to establish the comparative efficacy, safety, and optimal positioning of these therapies within the CSU treatment algorithm.

#### 5.4.2. Biomarkers, Endotypes, and Phenotypes in Treatment Selection

The expanding range of targeted therapies for CSU, together with considerable variability in treatment response and persistent inadequate disease control in a substantial proportion of patients, has increased interest in clinical and laboratory features that may help predict treatment response and support more individualized patient selection. This reflects the underlying phenotypic and endotypic heterogeneity of CSU.

Several clinical and laboratory parameters have been investigated as potential markers of CSU endotypes and treatment response, including total IgE, anti-TPO IgE and anti-TPO IgG antibodies, ASST results, basophil reactivity assessed by the basophil activation test (BAT) and/or basophil histamine release assay (BHRA), basopenia, and eosinopenia. In addition, clinical features such as atopic comorbidities have been investigated in relation to treatment response. However, the available evidence is not yet sufficient to allow individual biomarkers or CSU endotypes to reliably guide treatment selection. None of these parameters can currently be used as a stand-alone tool for therapeutic decision-making in routine clinical practice. They are therefore best regarded as supportive or predictive markers rather than direct determinants of treatment selection.

Most currently available biomarker-response data in CSU relate to omalizumab, reflecting the substantially longer clinical experience with anti-IgE therapy. Real-world evidence also suggests that the presence of atopic comorbidities may be associated with greater clinical improvement during dupilumab treatment, although this association requires further validation. Despite the expanding range of targeted therapies addressing distinct pathogenic pathways, clinically validated biomarkers that can reliably identify which patients are most likely to benefit from specific therapies remain limited. Moreover, the relationships between biomarkers, clinical phenotypes, and underlying endotypes remain insufficiently integrated, with these features often being evaluated separately rather than within a unified framework.

Table 2 summarizes the proposed relationships between CSU endotypes and phenotypes, candidate clinical and laboratory markers, potential associations with treatment response, potentially relevant targeted therapies, the nature and strength of supporting evidence, and their current clinical applicability. However, these associations should not be interpreted as a validated biomarker-guided treatment algorithm. Further identification and prospective validation of reliable markers may facilitate more rational treatment selection in the future. Endotype- and biomarker-guided management currently remains an evolving direction in precision medicine rather than an established standard of care in CSU [1,28,131,132,133].

From a practical perspective, the currently available evidence supports the use of endotypic and phenotypic features as contextual rather than prescriptive factors in therapeutic decision-making. A type I autoallergic pattern, particularly when accompanied by normal or elevated total IgE, is associated with a higher probability of an early response to omalizumab, whereas features suggestive of type IIb autoimmune CSU, including low total IgE, basophil or eosinophil depletion, positive basophil functional tests, and autoimmune comorbidities, are associated with a slower or less favorable response to omalizumab and may support consideration of alternative therapeutic strategies. Cyclosporine appears to be more effective in some patients with type IIb autoimmune features, although its safety profile limits long-term use.

Dupilumab may be particularly attractive when CSU coexists with other type 2 inflammatory diseases, whereas remibrutinib provides an oral targeted option with rapid clinical activity and a mechanism affecting both FcεRI-dependent effector-cell activation and B-cell signaling. Importantly, however, no clinical or laboratory profile has yet been prospectively validated to preferentially select remibrutinib, dupilumab, or other emerging targeted therapies. Thus, treatment selection should currently integrate disease activity, previous treatment response, comorbidities, safety considerations, route-of-administration preferences, and patient preference together with, rather than be determined by, candidate endotypic biomarkers.

## 6. Conclusions and Perspectives

Chronic spontaneous urticaria remains a disease with a complex and incompletely understood pathogenesis, posing significant therapeutic challenges. However, recent years have witnessed a rapid development of targeted therapies, gradually transforming existing treatment approaches. The introduction of omalizumab ushered in the era of biologic therapy for CSU, and subsequent agents, such as dupilumab and BTK inhibitors, are expanding therapeutic options, particularly in patients with inadequate disease control.

The increasing understanding of CSU immune endotypes provides a biological framework for the future development of more precise therapeutic strategies. Candidate biomarkers and endotypic features may help characterize patterns of treatment response; however, their routine use for therapeutic selection remains limited by the lack of standardized cut-off values, variable assay availability, and insufficient validation in large prospective cohorts. Current evidence therefore does not support a validated biomarker- or endotype-driven treatment algorithm. At present, treatment decisions should integrate disease activity, previous treatment response, comorbidities, safety considerations, route of administration, and patient preferences, with endotypic biomarkers regarded as supportive rather than prescriptive factors.

Promising data on new drug classes, such as BTK inhibitors, anti-c-KIT antibodies, and therapies targeting other components of the immune response, suggest the potential to achieve effective and potentially more durable disease control. Whether these effects translate into true disease modification remains uncertain and requires confirmation in long-term prospective studies. At the same time, further studies are needed to assess the durability of treatment response, safety, and the optimal positioning of emerging therapies within the therapeutic algorithm. Differences in study design, patient populations, treatment duration, and outcome measures currently limit direct comparisons between emerging therapeutic classes. Importantly, future research should also clarify how emerging therapies can be integrated into real-world treatment pathways and whether specific patient subgroups can be reliably matched to particular targeted interventions.

In the future, the integration of clinical, immunological, and molecular data to improve phenotyping and endotyping of patients with CSU may be crucial. Importantly, biomarkers, clinical phenotypes, and underlying endotypes should be evaluated within an integrated framework rather than as isolated features. The insufficient integration of these domains currently represents an important evidence gap and may limit the translation of emerging biomarker findings into clinically useful treatment strategies. Such an integrated approach may ultimately support more rational therapy selection, although prospective validation of biomarker-guided strategies remains necessary.

The management of chronic spontaneous urticaria is increasingly moving toward a more personalized, mechanism-based approach. Nevertheless, precision medicine in CSU remains an evolving concept rather than a fully implemented clinical reality. Further development of therapies targeting specific pathogenetic mechanisms, combined with advances in biomarker identification and validation, may significantly improve the prognosis and quality of life of patients with CSU in the coming years. Future progress will depend on establishing clinically useful biomarkers, validating endotype-treatment associations, addressing unresolved controversies and evidence gaps, and integrating evidence from comparative and long-term studies into treatment algorithms.

## Figures and Tables

**Table 1:** Overview of Mechanism-Based Targeted and Advanced Therapies in Chronic Spontaneous Urticaria.

Drug/Class	Target/Pathway	Clinical Status	Key Evidence/Role	Main Limitations	References
Omalizumab	Free IgE and FcεRI downregulation	Approved biologic therapy for CSU in patients aged ≥12 years; established second-line option.	Clinical trials and real-world studies support the efficacy and favorable safety profile of omalizumab, with responses reported in about 45% of antihistamine-refractory patients and complete disease control in approximately 30%. Patients with type I autoallergic CSU and higher baseline total IgE may respond particularly well.	A substantial proportion of patients do not achieve complete disease control. Low total IgE and autoimmune features may be associated with poorer or delayed response. [67,68,69,70,71,72,73,80]	[34,55,56,57,58,59,60,61,62,63,64,65,66,67,68,69,70,71,72,73,74,75,76,77,78,79,80,81]
Emerging anti-IgE therapies and omalizumab biosimilars	IgE pathway	Ligelizumab showed limited further development in CSU after phase III results; UB-221 and lesigercept/YH35324 remain under investigation; omalizumab biosimilars are approved or under development depending on region.	Ligelizumab showed promising phase II results but did not demonstrate superiority over omalizumab in PEARL 1 and 2. UB-221 and lesigercept/YH35324 represent alternative IgE-targeting strategies.	The clinical role of investigational agents remains uncertain. Access to and positioning of biosimilars may vary by region.	[82,83,84,85,86,87,88,89,90,91]
Dupilumab	IL-4Rα blockade inhibiting IL-4 and IL-13 signaling	FDA-approved for CSU in patients aged ≥12 years; EMA recommended extension of the indication to children aged 2-12 years.	LIBERTY-CSU CUPID-A and CUPID-C showed significant reductions in disease activity, pruritus severity, and urticaria activity in antihistamine-refractory, omalizumab-naive patients. Dupilumab may be relevant in patients with omalizumab intolerance, unavailability, or comorbidities associated with type 2 inflammation.	CUPID-B did not meet the primary endpoint in patients with omalizumab intolerance or inadequate response to anti-IgE therapy, suggesting involvement of mechanisms independent of type 2 inflammation in this subgroup.	[21,51,92,93,94,95,96,97,98]
BTK inhibitors: remibrutinib, rilzabrutinib, fenebrutinib, TAS5315	BTK-dependent FcεRI/BCR signaling	Remibrutinib is an FDA-approved oral option for adults with antihistamine-refractory CSU and has received marketing authorization in the European Union. Other BTK inhibitors discussed remain investigational in CSU.	Remibrutinib showed significant improvement in UAS7 in REMIX 1 and REMIX 2, with effects maintained through week 24 and sustained efficacy up to 52 weeks. Rilzabrutinib showed rapid improvement in phase II and reduced IgG autoantibodies, including anti-TPO and anti-FcεRI. The involvement of BTK in both FcεRI- and B-cell-mediated signaling provides a mechanistic rationale for activity across different CSU pathogenic pathways, although endotype-specific clinical efficacy has not yet been established.	Long-term placement in the therapeutic algorithm requires further clarification. Fenebrutinib was discontinued due to adverse events including elevated liver enzyme levels. Rilzabrutinib and TAS5315 remain under investigation in CSU.	[41,99,100,101,102,103,104,105,106,107,108,109,110,111,112,113,114,115]
c-KIT inhibitors: barzolvolimab and briquilimab	c-KIT signaling and SCF-dependent mast cell survival	Barzolvolimab is being evaluated in phase III EMBARQ-CSU 1 and EMBARQ-CSU 2; briquilimab is being evaluated in the phase 1b/2a BEACON study.	Barzolvolimab produced early and marked reductions in disease activity in both omalizumab-experienced and omalizumab-naive patients and was generally well tolerated. c-KIT blockade directly targets mast cell survival.	Long-term efficacy and safety require confirmation. Reported adverse events included neutrophil count decrease and hair color changes.	[39,40,116,117,118,119,120]
MRGPRX2 pathway inhibitors: EP262 and EVO756	MRGPRX2-mediated mast cell activation	EP262 development was halted; EVO756 is currently in phase II clinical trials.	MRGPRX2-mediated mast cell activation may represent an additional therapeutic target in CSU.	EP262 studies were halted due to safety concerns. Data on EVO756 are currently insufficient to evaluate efficacy.	[37,38,121,122]
Tezepelumab	TSLP and type 2 inflammatory signaling	Approved for severe asthma and chronic rhinosinusitis with nasal polyps; investigated in CSU.	The phase IIb INCEPTION study did not meet its primary endpoint. Subgroup analyses suggested beneficial effects in patients previously untreated with anti-IgE therapy, particularly those with lower baseline IgE and longer disease duration.	Tezepelumab did not demonstrate superiority over placebo in the primary analysis, and its role in CSU remains uncertain.	[123,124,125]
Secukinumab	IL-17A inhibition	Evaluated in a small clinical study of severe refractory CSU.	In eight patients unresponsive to H1-antihistamines and omalizumab, all patients showed significant improvement, although the therapeutic effect developed gradually.	Evidence is limited to a small clinical study and requires further confirmation.	[42]
Mepolizumab, Benralizumab	IL-5 pathway and eosinophilic inflammation	Evaluated in clinical studies in CSU.	Benralizumab did not demonstrate significant clinical benefit over placebo in the phase IIb ARROYO trial. Mepolizumab was evaluated in an open-label, single-arm exploratory study in antihistamine-refractory CSU and was not associated with clinical or laboratory improvement.	Evidence remains limited and differs between agents. The findings for benralizumab and the exploratory design of the mepolizumab study indicate that the therapeutic role of IL-5 pathway inhibition in CSU remains uncertain.	[43,126,127]

Abbreviations: anti-TPO, anti-thyroid peroxidase antibodies; BCR, B-cell receptor; BTK, Bruton’s tyrosine kinase; c-KIT, stem cell factor receptor/CD117; CSU, chronic spontaneous urticaria; EMA, European Medicines Agency; FDA, U.S. Food and Drug Administration; FcεRI, high-affinity IgE receptor; IgE, immunoglobulin E; IgG, immunoglobulin G; IL, interleukin; IL-4Rα, interleukin-4 receptor alpha; MRGPRX2, Mas-related G protein-coupled receptor X2; SCF, stem cell factor; TSLP, thymic stromal lymphopoietin; UAS7, weekly Urticaria Activity Score.

**Table 2:** Endotype- and Phenotype-Associated Biomarkers and Their Potential Relevance to Treatment Response and Therapeutic Selection in CSU.

CSU Endotype/Phenotype	Biomarker/Clinical Feature	Reported/Potential Association with Treatment Response	Potentially Relevant Targeted Therapy	Strength of Evidence	Current Clinical Applicability	References
Type I autoallergic CSU/Type IIb autoimmune CSU	Baseline total serum IgE	Higher baseline total IgE has been associated with a more favorable and/or earlier response to omalizumab, whereas low baseline IgE has been associated with poorer or delayed response.	Omalizumab	Supported by multiple observational studies and meta-analyses; baseline total IgE has shown potential predictive value for omalizumab response, although no reliable threshold for predicting nonresponse has been established and further validation in diverse patient populations is needed.	**Endotypic relevance:** Normal or elevated total IgE is more frequently observed in type I autoallergic CSU, whereas low total IgE is a supportive feature of type IIb autoimmune CSU. **Treatment relevance:** Baseline total IgE may help predict omalizumab response, but low IgE should not be used to exclude patients from treatment, and no validated IgE cut-off is available for treatment selection. **Overall limitation:** Total IgE is insufficient as a stand-alone criterion for endotype assignment or treatment selection.	[31,68,69,72,73,134,135,136,137,138,139]
Type I autoallergic CSU	IgE autoantibodies against thyroperoxidase (TPO) (anti-TPO IgE)	Patients with anti-TPO IgE-positive CSU demonstrated a significant clinical response to omalizumab compared with placebo.	Omalizumab	Supported by a multicenter randomized, double-blind, placebo-controlled trial in anti-TPO IgE-positive patients; however, the independent predictive value of anti-TPO IgE was not established.	Potential endotypic biomarker of type I autoallergic CSU; however, its role in routine treatment selection has not been established.	[76]
Type IIb autoimmune CSU	Anti-TPO IgG and anti-TPO IgG/total IgE ratio	Elevated anti-TPO IgG is associated with the type IIb autoimmune profile and has been linked to poorer omalizumab response in a real-world study. However, its predictive value has not been consistently demonstrated across studies. A higher anti-TPO IgG/total IgE ratio has been associated with poorer response and has also been identified as an independent predictor of the speed of response.	Omalizumab	Supported by the multicenter PURIST endotype study and several retrospective observational studies, including real-world data; overall evidence is suggestive but heterogeneous, and further validation is required.	Supportive marker of the type IIb autoimmune profile and potential predictor of omalizumab response; combined interpretation of anti-TPO IgG with total IgE may provide additional predictive information, but neither measure is validated as a stand-alone tool for treatment selection.	[31,140,141,142]
Type IIb autoimmune CSU	Positive autologous serum skin test (ASST)	ASST positivity has been associated with a slower response to omalizumab.	Omalizumab	Supported by retrospective observational studies and a small prospective study showing an association between ASST positivity and delayed omalizumab response; larger prospective studies are needed to confirm its predictive value.	Supportive test for autoimmune/type IIb features and a potential predictor of response kinetics; not sufficient as a stand-alone basis for treatment selection.	[31,35,139,143]
Type IIb autoimmune CSU	Positive basophil activation test (BAT) and/or basophil histamine release assay (BHRA)	Positive basophil reactivity has been associated with poorer and/or delayed response to omalizumab; BHRA positivity has also been linked to the need for treatment intensification or discontinuation due to inadequate response.	Omalizumab	Supported by endotype-focused studies, observational data, and a systematic review; BAT/BHRA are well-supported functional markers of type IIb autoimmune CSU, whereas their predictive value for treatment response requires further validation.	Useful functional tests for identifying type IIb autoimmune CSU and characterizing a more active/severe disease profile; however, further standardization is needed to support broader implementation in routine clinical practice.	[31,144,145,146]
Type IIb autoimmune CSU	Basopenia	Basopenia has been associated with poorer and/or slower response to omalizumab.	Omalizumab	Supported by observational and mechanistic studies; basopenia is associated with autoimmune/type IIb features and higher disease activity, although its independent predictive value requires further validation.	Readily accessible supportive marker of the type IIb autoimmune profile and disease activity; may provide additional prognostic information regarding omalizumab response, but is not sufficient as a stand-alone basis for treatment selection.	[147,148,149,150,151]
Type IIb autoimmune CSU	Eosinopenia	Eosinopenia has been associated with poorer response to omalizumab.	Omalizumab	Supported by observational studies; eosinopenia is associated with type IIb autoimmune features, higher disease activity, and poorer treatment response, although its predictive value requires further validation.	Readily accessible supportive marker of the type IIb autoimmune profile and disease activity; may provide additional prognostic information regarding omalizumab response, but is not sufficient as a stand-alone basis for treatment selection.	[151,152]
Atopic/type 2 inflammatory phenotype	Atopic comorbidities (atopic dermatitis, asthma, and/or allergic rhinoconjunctivitis)	The presence of atopic comorbidities has been associated with greater clinical improvement during dupilumab treatment. Dupilumab has demonstrated effective disease control in patients with difficult-to-treat CSU, including most omalizumab-experienced individuals.	Dupilumab	Supported by a multicentre ambispective real-world cohort study; the association was independently observed in multivariable analysis but requires confirmation in larger prospective cohorts.	Potentially useful for identifying patients with atopic comorbidities who may derive greater benefit from dupilumab, but not yet validated as a stand-alone biomarker for treatment selection.	[133]

Abbreviations: ASST, autologous serum skin test; BAT, basophil activation test; BHRA, basophil histamine release assay; CSU, chronic spontaneous urticaria; IgE, immunoglobulin E; anti-TPO IgE, immunoglobulin E autoantibodies against thyroid peroxidase; anti-TPO IgG, immunoglobulin G autoantibodies against thyroid peroxidase.

## Data Availability

No new data were created or analyzed in this study. Data sharing is not applicable to this review.
